# Automatically Generated Algorithms for the Vertex Coloring Problem

**DOI:** 10.1371/journal.pone.0058551

**Published:** 2013-03-13

**Authors:** Carlos Contreras Bolton, Gustavo Gatica, Víctor Parada

**Affiliations:** 1 Departamento de Ingeniería Informática, Universidad de Santiago de Chile, Santiago, Chile; 2 Escuela de Informática, Universidad Andrés Bello, Santiago, Chile; Albert Einstein College of Medicine, United States of America

## Abstract

The vertex coloring problem is a classical problem in combinatorial optimization that consists of assigning a color to each vertex of a graph such that no adjacent vertices share the same color, minimizing the number of colors used. Despite the various practical applications that exist for this problem, its NP-hardness still represents a computational challenge. Some of the best computational results obtained for this problem are consequences of hybridizing the various known heuristics. Automatically revising the space constituted by combining these techniques to find the most adequate combination has received less attention. In this paper, we propose exploring the heuristics space for the vertex coloring problem using evolutionary algorithms. We automatically generate three new algorithms by combining elementary heuristics. To evaluate the new algorithms, a computational experiment was performed that allowed comparing them numerically with existing heuristics. The obtained algorithms present an average 29.97% relative error, while four other heuristics selected from the literature present a 59.73% error, considering 29 of the more difficult instances in the DIMACS benchmark.

## Introduction

The vertex coloring problem (VCP) consists of identifying the lowest number of colors required to color a graph. Let *G* = (*V*, *E*) be an undirected graph, where *V* is a set of vertices and *E* is a set of edges. A function *F*: *V* ↦ <$>\raster="rg1"<$> is defined, where <$>\raster="rg1"<$> is a finite set of colors such that if *u* and 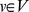 and 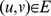, then 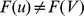 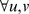. The objective is to find the chromatic number, 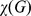, corresponding to the minimum number of colors needed to color *G* [1].

Because of its NP-hardness [2], the VCP has been treated using various methods: exact, constructive heuristics, and metaheuristics [3]. The exact methods consider different integer programming formulations. Its solution, however, does not completely consider the problem as certain instances belong to the known benchmarks that cannot be solved in reasonable times and their 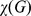 values remain unknown. To obtain results with low computational times, Méndez-Díaz and Zabala [4], [5] combine linear integer programming with instance preprocessing methods, while Malagutti et al. [6] combine branch-and-price with an algorithm that integrates tabu search, greedy, and evolutionary algorithm components and reports 33 optimum values, of which five were “open for several years”, leaving 28 problems with unknown 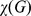 among the 115 analyzed instances.

Constructive heuristic methods integrate elementary heuristic functions that color a graph step-by-step, considering decisions on the next vertex to color and color to use [7]–[9]. Because the heuristics can be easily implemented in polynomial time, they are also used as tools within other algorithms. Malaguti et al. [10] propose an evolutionary algorithm that combines tabu search, generating a set of initial solutions through the well-known greedy constructive heuristics SEQ [3], DSATUR [7] and Recursive Largest First (RLF) [8]. The initial solutions are then improved by solving a set-covering problem. Although such hybridization obtains better solutions than other methods, several hours of computational time are required to achieve them.

Various metaheuristic approaches have resulted from heuristic combinations to solve the VCP. Combining evolutionary techniques with simulated annealing has allowed finding new chromatic numbers for various test instances [11], [12]. Another interesting combination of a genetic algorithm with the classical tabu search [13] provides a method with five different versions, obtaining optimum solutions for some hard instances of the DIMACS benchmark, with a computational time limit of 12 hours [14]. The existing VCP results indicate that an adequate method combination can solve some of the more difficult instances, but the combinations revised so far represent a small part of those that can possibly be revised.

The selection of an appropriate combination of heuristics to solve a problem, can be performed automatically. In fact, Minton [15] proposed an analytical learning system, which from a set of generic heuristics, produces specific heuristic combinations for the constraint-satisfaction problem. For the same problem, Epstein et al. [16] have proposed an adaptive engine that, from a set of elementary methods, produces a combination of heuristics that learn from their past decisions, as the search progresses. The engine is not only capable of finding novel heuristic for the problem, but also it rediscovers existing heuristics. Also, Nareyek [17] proposed a method that uses reinforcement learning to discover the best heuristic to be adopted at each stage of a search process. A particular case of the constraint satisfaction problem is the MAX-SAT, which is to determine a solution that satisfies a maximum number of constraints, therefore, it can be seen as a typical combinatorial optimization problem. Bain et al. [18] proposed a novel way to solve the problem by means of genetic programming [19], [20], i.e., evolving algorithms that are combinations of elementary heuristics already known for the problem. Also, Fukunaga [21], [22] proposed a heuristic evaluation system for solving the satisfiability problem. The new heuristics produced are competitive with some of the existing ones, showing that for this problem, genetic programming can automatically produce new algorithms. In the field of combinatorial optimization, the hyper-heuristics [23] are a generalization of metaheuristics. Such techniques also can combine heuristics to solve difficult optimization problems. These have been tested with various problems such as timetabling [24] problem and strip packing problem [25].

In this paper, we suggest that a combination of heuristics for VCP can be performed using evolutionary computation, which allows new algorithms to be produced that use already existing techniques constructed from elementary heuristic components and control structures typical of any algorithm type, producing procedures that can compete with existing heuristics. The participation of the elementary heuristics and control structures are coded using binary *strings* that are subjected to selection, crossover and mutation operations at each stage [26], [27]. Using some control parameters, we detected that the new algorithms have characteristics common to existing heuristics.

The automatic revision of heuristic combinations enables studying the potential algorithms that solve the VCP with low computational requirements and obtain high-quality solutions. The algorithms that can be generated would thus allow detecting heuristic combinations with good performance for given groups of VCP instances.

The following section of this paper describes the procedures involved in generating the new algorithms, while the third section presents and discusses the computational results of the generated algorithms. The last section presents the conclusions of the study.

### Procedure for Generating the Algorithms

This section presents the evolutionary process used to obtain algorithms for the VCP. The process follows similar steps to those used in genetic programming to represent a problem [19], [20]. This means that it has to be defined the representation used, the data structures, the fitness function definition, the corresponding functions and terminals with elementary heuristics used to generate algorithms, the testing and validation instances, the parameter calibration procedure, and the hardware and software used.

### Evolutionary Process

**Figure 1:**
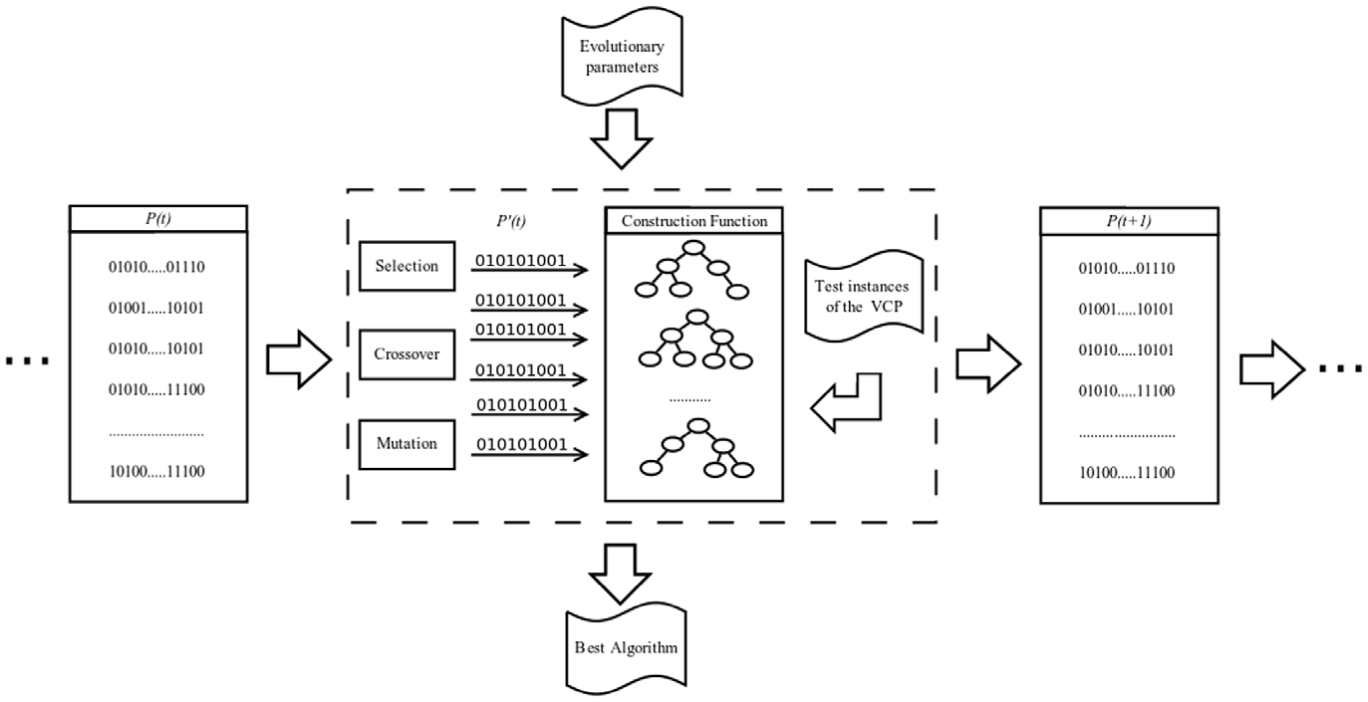
Evolutionary process.

To perform algorithm evolution, we designed and implemented an evolutionary algorithm that operates in a genotype-phenotype mode [28]. As genotype, we use a structure that specifies the participation of each heuristic component and typical instructions, and as phenotype, a tree structure that corresponds to the algorithm to be constructed. The evolution of a population with fixed size is performed using binary tournament selection operators between 2 individuals selected randomly, two-point crossover, and binary mutation [27]. Each new population contains binary strings obtained from the current population. Every time a string is generated, the fitness function evaluates the individual, constructs an instructions tree and validates its performance on a set of test instances. Figure 1 describes the evolutionary algorithm scheme, showing the stages involved in the generation of population *P*(*t* +1) from population *P*(*t*).

The evolutionary process considers 10 executions of 12 problem groups: DSJ, LEI, GOM, MYC, HOS, KOZ, CAR, LAT-SCH, SGB, REG, MYZ and CUL, available at http://mat.gsia.cmu.edu/COLOR/instances.html. Consequently, 120 evolutionary processes are performed with populations of 50 individuals and a stopping criterion of 100 generations. To decrease the computational time used in the implementation, the fitness function is evaluated in parallel, considering 6 processors with shared memory. The evaluations are distributed among the parallel processors from a waiting queue.

### Representing the Genotype and Phenotype

**Figure 2:**
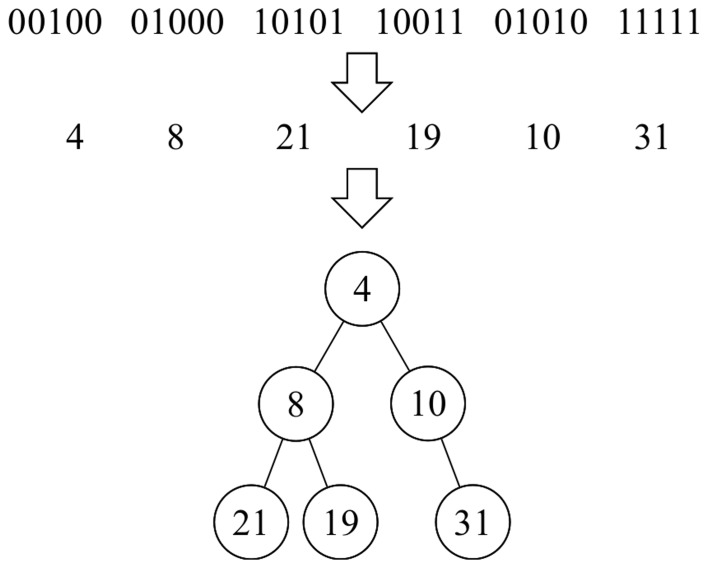
Decoding a binary string.

The elementary components of the artificially generated algorithms are identified using an integer number that is transformed into a binary number. Five bits are thus used to represent 32 different components. A binary string indicates the components and the order in which they are selected to construct the corresponding artificial algorithm (phenotype), represented using a preordered binary tree [29]. Figure 2 describes a string with 30 bits and a binary tree with six nodes.

### Data Structure

The data structure from which the artificial algorithms are created stores the graph to be colored. The graph is stored in an adjacent list. Implementation uses the Boost Graph Library [30], which contains functions optimized for recurrent operations on graphs.

### Fitness Function

We define a fitness function containing three terms: the first measures the coloring degree of the graph, the second represents the number of colors used, and the third corresponds to the tree size.

Let 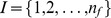 be a set of instances in the VCP, and let us introduce the following definitions:


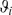
: number of colors used to color graph *i*,

*h*: tree depth,


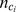
: number of vertices with a color assigned to graph *i*,


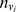
: total number of vertices in graph *i*,


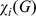
: best known chromatic number for graph *i*,


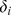
: number of nodes in algorithm *i*.

Let 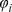 be the relative error of the number of colored vertices with respect to the number of vertices in problem *i* (Eq. 1).
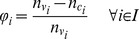
(1)


Equation 2 corresponds to the fitness function. The first internal term of the summation represents the average relative error of the number of colors used for all instances, the second weights the average deviation of the best chromatic number known, and the third weights a deviation of the tree size for a height defined by *h*. The values of *f*(*G*) are in the [0,1] range. The terms are weighted parametrically by values *a*, *b* and *c*.
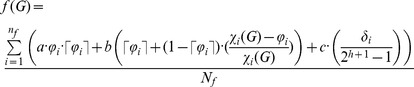
(2)


### Function and Terminal Definitions

As elementary components of the produced algorithms, function and terminal sets are defined. Executing a function requires one or two arguments that can also be functions or terminals, denoted *function(argument1,argument2)*. Each graph vertex is numbered, as are the colors available for coloring. The functions are subdivided into coloring and generic functions. The former assign a color to the vertex based on a defined criterion and require a terminal that returns a vertex as an argument, while the latter receive a Boolean value as an argument that corresponds to the evaluation of a subtree.

### Coloring Functions

- **Greedy (v):** It assigns the least feasible color to vertex *v*. When changing the vertex color, the function returns the value true, otherwise it returns false. It is based on the *Iterated Greedy* algorithm [9].
- **More-frequent-color(v):** It assigns the feasible color with higher frequency in the graph to vertex *v*. If this is not possible, it assigns a color to the vertex using *Greedy* (*v*). If the function changes the color assigned to the vertex, it returns true; otherwise, it returns false.
- **Less-frequent-color(v):** It assigns a less frequent feasible color in the graph to vertex *v*. If this is not possible, it assigns a feasible color that does not generate conflicts using *Greedy* (*v*). If the function changes the color assigned to the vertex, it returns true; otherwise, it returns false.
- **Swap-color(v_1_,v_2_):** It exchanges the color of vertex *v*_1_ with that of vertex *v*_2_. If no conflicts are generated, it returns true; otherwise, it returns false.
- **Greedy-adjacents(v):** It assigns a feasible color to the vertices adjacent to *v* using *Greedy*(*v*) and returns the value true if it assigns at least one color to an adjacent vertex; otherwise, it returns false.
- **Uncoloring(v):** It removes the current color of *v*, returning the true value; if *v* has no color assigned, it returns false.
- **Uncoloring-adjacents(v):** It removes the color of each vertex adjacent to *v* and returns a true value if removing at least one color is feasible; otherwise, it returns false.

### Generic Functions

**Figure 3:**
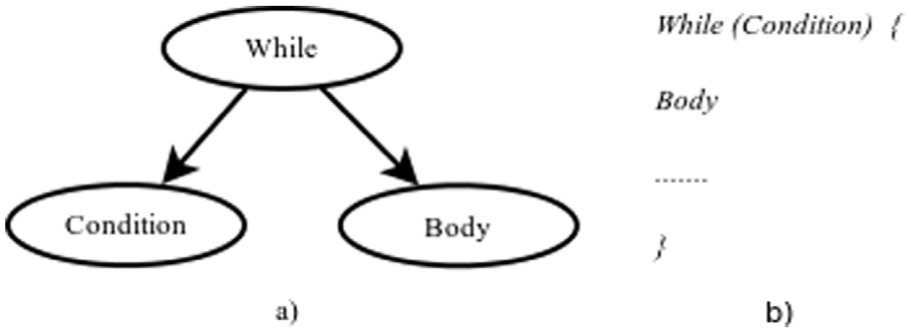
While cycle and its equivalence in pseudocode.

A set of generic functions, i.e., those that participate in the algorithms as control structures, is defined; they are denoted using arborescent arguments: *Equal*(*P*_1_,*P*_2_), *And*(*P*_1_,*P*_2_), *Or*(*P*_1_,*P*_2_), and *Not* (*P*_1_,*P*_2_). The following two functions are also used:

- **If (P_1_,P_2_):** It executes *subtree P*_2_ if *subtree P*_1_ has a true value. It returns a true value if executing the second argument is feasible (regardless of its return); otherwise, it returns false.
- **While(P_1_,P_2_):** It performs a cycle on *subtree P*_2_ if the condition of *subtree P*_1_ remains true. It returns true when it modifies the graph color; otherwise, it returns false. *Subtree P*_1_ corresponds to the condition and *subtree P*_2_ to the body. The stop criterion is met when *P*_1_ returns false, or when a number of iterations (10) elapses without any change in the data structure or when it is exceeded a predefined maximum number of iterations equal to number of vertices of the instance. Figure 3*a* shows a tree with a cycle whose left offspring is the condition and right offspring the body. Fig. 3*b* shows its equivalent pseudocode.

### Set of Terminals

The terminals are subdivided into vertex identifiers and state indicators. The vertex identifiers aim to identify particular vertices in the graph. If no vertex is found that fulfills the desired characteristic, it returns false. Conversely, the latter return true or false, and they are used as arguments of the condition of generic functions.

### Vertex Searching Terminals

- **First-vertex:** It identifies the lowest numbered vertex, uses the First-Fit [31] vertex selection criterion, and returns false if it does not find a vertex with that characteristic.
- **More-frequent-color-vertex:** It identifies the highest numbered vertex with the most frequently used color in the graph; if it does not exist, it returns false.
- **Less-frequent-color-vertex:** It identifies the lowest numbered colored vertex with a less frequent color; if it does not exist, it returns false.
- **Lowest-numbered-color-vertex:** It identifies the highest numbered vertex with the lowest numbered color; if it does not exist, it returns false.
- **Highest-numbered-color-vertex:** It identifies the highest numbered vertex with the highest numbered color; if it does not exist, it returns false.
- **Minimum-degree-vertex:** It identifies the lowest numbered vertex with the fewest adjacent vertices, based on the Minimum Degree Ordering [32] vertex selection criterion; if the vertex does not exist, it returns false.
- **Largest-degree-vertex:** It identifies the vertex with the most adjacent vertices. If there is more than one vertex that fulfills this characteristic, it returns the lowest numbered vertex that fulfills this condition, based on the Modified Largest Degree Ordering [31] vertex selection criterion; if the vertex does not exist, it returns false.
- **Saturation-degree-vertex:** It identifies the vertex with the most adjacent vertices with different assigned colors, based on the Saturation Degree Ordering vertex selection criterion used by the *DSATUR* algorithm. If more than one vertex has this characteristic, it returns the lowest numbered vertex; otherwise, it returns false.
- **Incidence-degree-vertex:** It identifies the lowest numbered vertex with the most colored adjacent vertices, based on the Incidence Degree Ordering [31] vertex selection criterion; if it does not find a vertex, it returns false.
- **More-uncolored-adjacents-vertex:** It identifies the lowest numbered vertex with the most uncolored adjacent vertices, according to the vertex selection criterion of the RLF algorithm; if it does not find a vertex, it returns false.

### Boolean Terminals

These are variables that indicate the coloring state of the graph at any construction stage. The first, *Not-increase,* indicates if there is an increase of the number of colors used to color the graph. The other variable, *Exist-uncolored-vertex*, indicates if uncolored vertices remain in the graph.

### Adaptation and Test Cases

The problems used to test and adapt the individuals comprise a problem set from the DIMACS benchmark, which compiles instances considered as a standard set for the VCP [33].

### Parameter Calibration

The evolutionary algorithms’ computational performance has a direct relationship with the parameters used in the experimental stage. The parameters involved are the probabilities of crossover and mutation, the number of generations, the chromosome length and the parameters *a*, *b* and *c* of the fitness function. Based on the studies by Grefenstette [34], the crossover probability is set to 0.9. The mutation probability was fine-tuned with the following values: {0.005, 0.01, 0.02, 0.04, 0.08, 0.10, 0.12, 0.14, 0.16, 0.18, 0.20}, and it delivered a value of 0.16. In preliminary experiments, no improvements are detected after 100 iterations; the detention criterion of the evolutionary algorithm is thus set to 100 generations. In contrast with other problems approaches using evolutionary algorithms, where chromosome length is automatically defined by considering the problem to solve as the basis, e.g., in the travelling salesman problem, which is based on the number of cities [35], for this approach we must determine the chromosome length parameter. A fine-tuning is thus performed using the values {40, 80, 160, 320}, which delivers the value 160. Finally, the weights of the fitness function were adjusted in preliminary tests to give greater importance to the minimization of the error; the values adopted were: *a* = 0.3, *b* = 0.6 and *c* = 0.1.

### Hardware Used

A computer cluster was used, containing two machines with *Intel Xeon* E5045 2 *GHz* processors with 16 GB *RAM*, and six other machines with *AMD Opteron* 2210 1.8 *GHz* processors with 2 GB *RAM*. All used the *GNU/Linux Ubuntu* 10.10 distribution.

## Results and Discussion

**Figure 4:**
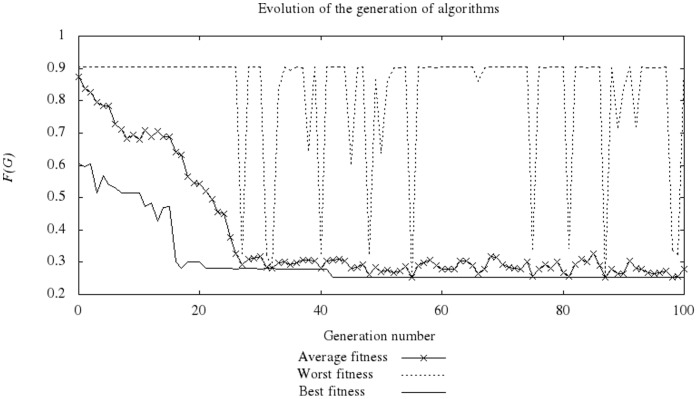
Evolving VCP algorithms.

**Figure 5:**
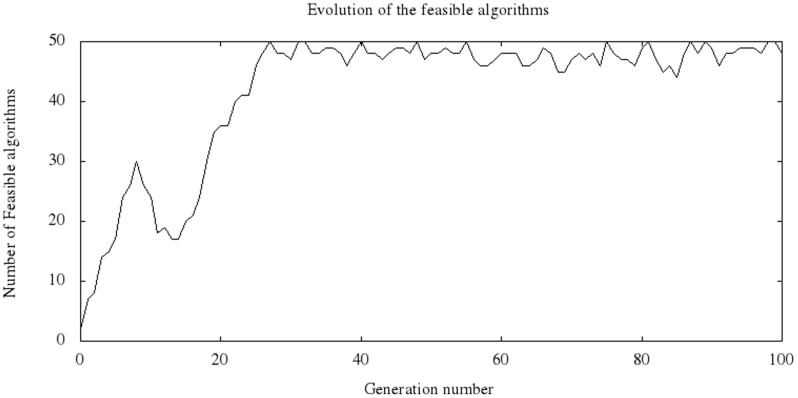
Evolution of feasible algorithms.

As the evolutionary process advances, increasingly better individuals are generated until reaching the final stages, where there is a convergence to populations with individuals of diverse quality; most of these populations have a fitness value close to the best value of the population. Figure 4 shows a typical case of the evolutionary process, considering 5,000 individuals that correspond to one of the 10 runs made on a problem group. The ordinate shows the fitness value, and the abscissa indicates the generation number. In this case, the randomly generated population had fitness values approximately between 0.6 and 0.9; in the first generations, there is already a strong decrease in the fitness value of the best individual of each population. Starting in generation 27, the average value and the value of the best individual are close to one another. The best individual obtained in this run was found in generation 42. The curve of the worst individual during the process shows an individual with a fitness value close to 0.9 in many generations, which corresponds to individuals that do not color the graph correctly, according to our fitness definition, and therefore have a positive penalty value. However, their presence is detected with increasingly lower frequencies because the number of individuals that correctly color each graph typically increases, following the trend in Figure 5 for the same run.

**Figure 6:**
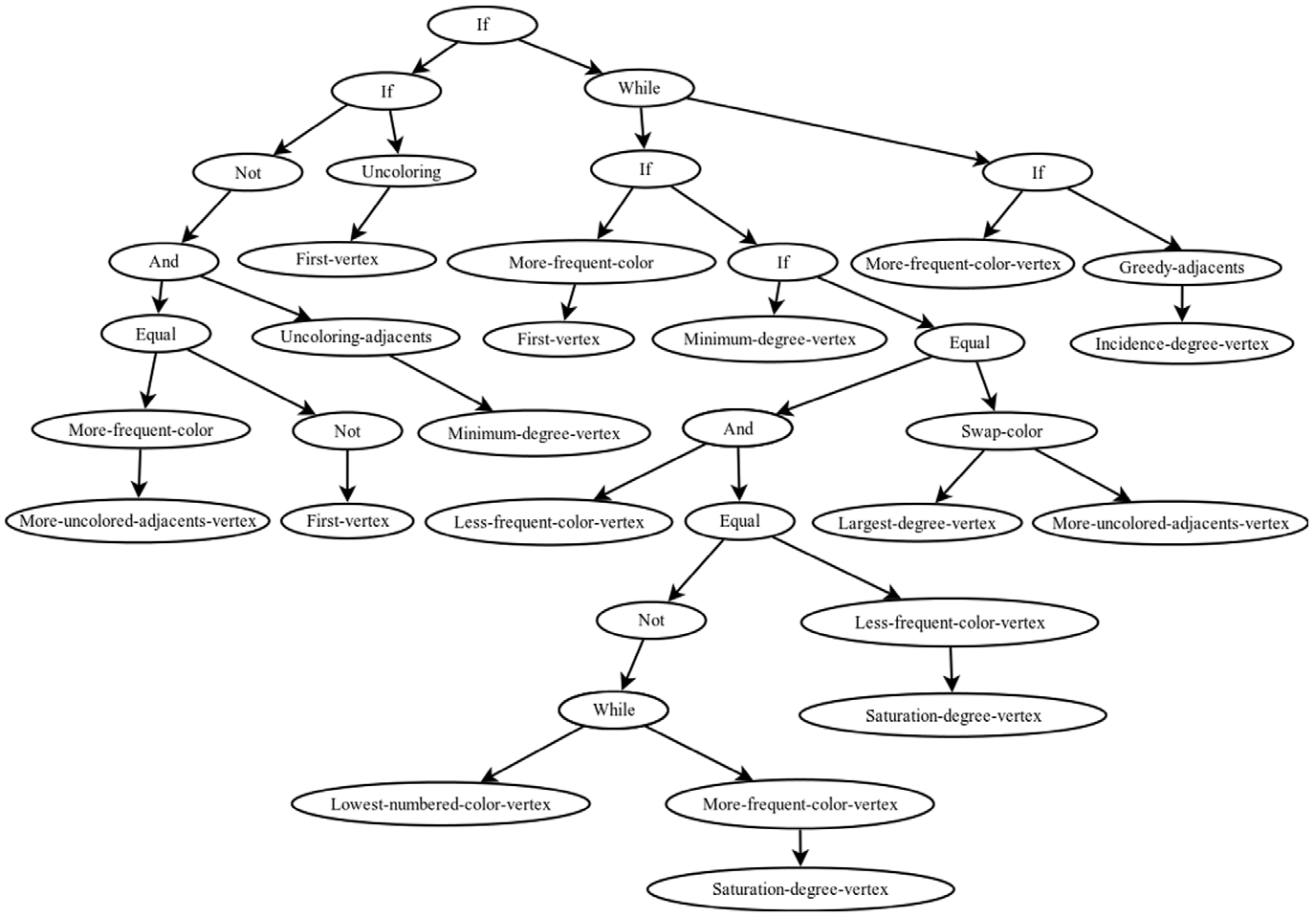
Representation tree of algorithm A3.

The different individuals generated during the evolutionary process can be manually decoded from their tree structure into their corresponding algorithms, described in pseudocode. The computational experiment was carried out using *h* = 9 in Eq. 2, thus ensuring that we obtain individuals with a legible and decodable structure in their algorithms. Algorithm 1 presents an illustrative case, which is obtained from the individual shown in Figure 6; its nodes correspond to the defined functions and terminals, and the arcs of each function or terminal define how the procedure works. According to the defined functions and terminals, Algorithm 1 is a constructive and greedy algorithm because it gradually colors the graph every time it applies a color and tries to not increase the number of colors. In its main stage, the algorithm selects a vertex using a criterion similar to the DSATUR algorithm to color it with the most frequent color. It performs this task within a *while* cycle (instructions 3 to 5), and refines the solution found (instructions 6 to 10), either by coloring the remaining vertices or exchanging already assigned colors.

Algorithm 1: Pseudocode of A3

**Input:** Graph

**Output:** ColoredGraph

01: More-frequent-color (More-uncolored-adjacents-vertex)

02: **while** More-frequent-color (First-vertex) **do**

03: **while** Lowest-numbered-color-vertex **do**

04: More-frequent-color (Saturation-degree-vertex)

05: **end while**

06: Less-frequent-color (Saturation-degree-vertex)

07: Greedy-adjacents (Incidence-degree-vertex)

08: Swap-color (Largest-degree-vertex, More-uncolored-adjacents-vertex)

09: **If** More-frequent-color-vertex **then**

10: Greedy-adjacents (Incidence-degree-vertex)

11: **end if**

12: **end while**

13: **return** Graph Colored

**Figure 7:**
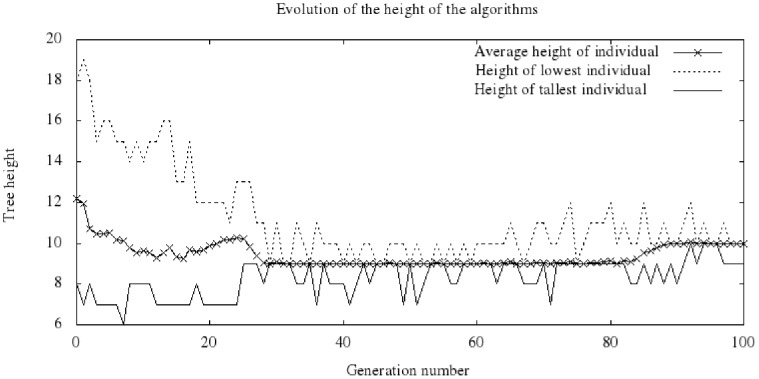
Evolution of the tree height.

The size of the individuals measured using tree height tends to stabilize at a value close to the height parameterized at the beginning of the evolutionary process. The initial randomly generated population determines a disperse range of possible heights for the individuals, and this range tends to decrease gradually as the evolutionary process occurs. Figure 7 illustrates a typical case, which corresponds to the same run presented in Figures 4 and 5. The figure shows the maximum, minimum and average height values in each population. Some stability can be identified in the individuals produced, with a value of 9, during 50 consecutive generations. This behavior is essentially due to the penalty considered in the fitness function.

**Figure 8:**
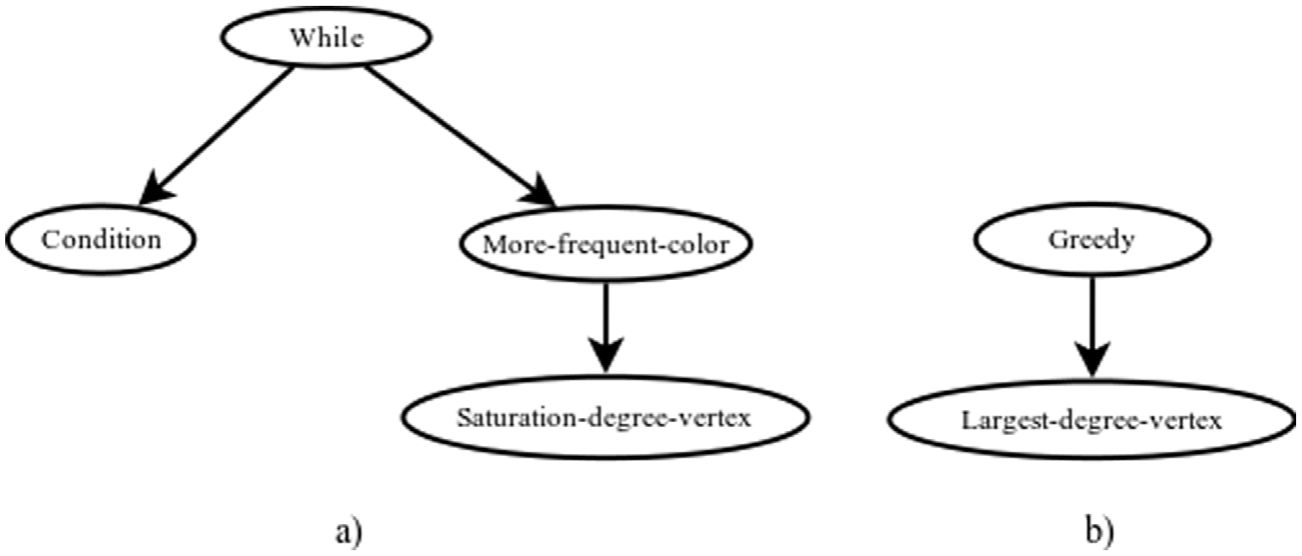
Repetitive constructions observed in the obtained individuals.

The various algorithms demonstrate the presence of common structures with characteristics similar to the existing heuristics for the VCP. Figure 8 shows two such structures. The first one (8a) corresponds to a greedy coloring of a vertex selected using the criterion of neighboring vertices with different colors, which is similar to the *DSATUR* heuristic algorithm; the second (8b) corresponds to a greedy coloring of the vertex with the highest degree, as in the Largest First algorithm [31].

The algorithms obtained through the evolutionary process are numerically competitive with elementary heuristics that solve the VCP. The generated algorithms find optimum solutions for some problems, as do existing heuristics. Table 1 presents the numerical results of three algorithms: the first produced with the DSJ instances, and the second and third with LEI. They are tested using 29 problems from the *DIMACS* benchmark. The first six columns correspond to the problem name, number of vertices, number of edges, density, optimum chromatic number, and best known chromatic number. The following four columns correspond to results obtained from heuristics presented in the literature: *Greedy Sequential*, *Largest First*, *Smallest First*, and *DSATUR,* which we consider as the conceptual bases for constructing our functions and terminals. The last six columns correspond to the chromatic number and computational time of our three algorithms, which we call A1, A2 and A3. The symbol “?” indicates that 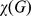 is unknown for the corresponding instance, and bold characters are used when the best known chromatic number was obtained.

**Table 1:** Numerical results.

Instance	*n*	*m*	*d(G)*	_χ(G)_	Best(χ)	*Greedy Sequential*	*Largest First*	*Smallest First*	*DSATUR*	A1		A2			A3		Best_*ϑ*_
										_*ϑ*_	Time (s)	_*ϑ*_	Time(s)	_*ϑ*_		Time(s)	
DSJC125.1	125	736	0.095	?	5	8	8	8	6	6	0.016	6	0.020	7		0.012	6
DSJC125.5	125	3891	0.502	?	17	26	24	29	22	22	0.073	22	2.890	23		2.921	22
DSJC125.9	125	6961	0.898	?	44	56	56	59	51	52	0.161	52	10.315	52		10.696	52
DSJR500.1	500	3555	0.028	12	12	15	15	16	13	13	0.142	13	0.205	**12**		0.099	**12**
DSJR500.1c	500	121275	0.972	?	85	109	107	121	90	93	12.613	93	30.524	93		18.536	93
DSJR500.5	500	58862	0.472	122	122	143	143	159	130	127	4.219	127	41.784	126		26.962	126
r125.1c	125	7501	0.968	46	46	51	50	51	46	**46**	0.241	**46**	11.497	**46**		10.735	**46**
r125.5	125	3838	0.495	36	36	44	44	47	38	39	0.091	39	9.305	39		11.222	39
r250.1	250	867	0.028	8	8	9	9	11	**8**	**8**	0.054	**8**	0.043	**8**		0.017	**8**
r250.1c	250	30227	0.971	64	64	76	77	78	65	67	1.827	67	14.843	67		20.321	67
r250.5	250	14849	0.477	65	65	79	79	84	68	69	0.580	69	14.803	67		10.519	67
r1000.1	1000	14378	0.029	20	20	26	26	28	**20**	**20**	1.339	**20**	2.985	**20**		1.620	**20**
r1000.5	1000	238267	0.477	234	234	275	276	317	250	243	36.791	245	51.879	252		34.181	243
le450_15a	450	8168	0.081	15	15	22	21	27	17	17	0.449	17	0.746	16		0.603	16
le450_15b	450	8169	0.081	15	15	22	22	27	16	17	0.452	16	0.734	17		0.573	16
le450_15c	450	16680	0.165	15	15	30	31	40	23	24	1.238	24	5.067	24		5.207	24
le450_15d	450	16750	0.166	15	15	31	30	37	24	24	1.241	24	5.135	25		5.594	24
le450_25a	450	8260	0.082	25	25	28	28	31	**25**	**25**	0.409	**25**	1.109	**25**		1.009	**25**
le450_25b	450	8263	0.082	25	25	27	27	34	**25**	**25**	0.391	**25**	0.971	**25**		0.889	**25**
le450_25c	450	17343	0.172	25	25	37	36	45	29	29	1.178	29	11.148	30		10.786	29
le450_25d	450	17425	0.172	25	25	35	35	43	28	29	1.205	29	11.181	28		10.663	28
le450_5a	450	5714	0.057	5	5	14	14	16	10	9	0.371	10	0.425	10		0.291	9
le450_5b	450	5734	0.057	5	5	13	13	18	9	10	0.363	10	0.428	10		0.281	10
le450_5c	450	9803	0.097	5	5	17	15	22	10	7	0.655	8	0.825	10		0.741	7
le450_5d	450	9757	0.097	5	5	18	17	24	12	7	0.654	7	0.823	6		0.827	6
anna	138	493	0.052	11	11	12	12	13	**11**	**11**	0.011	**11**	0.018	**11**		0.013	**11**
david	87	406	0.109	11	11	12	12	12	**11**	**11**	0.006	**11**	0.011	**11**		0.007	**11**

Of the 5,000 total algorithms revised in each evolution, an average of 83.47%, completely color the 12 evolution groups, i.e., approximately 500,810 algorithms feasible in the 120 executions are generated. The average computational time required to evolve the three algorithms from the 12 groups of instances used, considering the 10 executions for each group, is 43.85 hours for each algorithm. Two of the automatically generated algorithms obtain the optimum solution in 24.14% of the test problems, while the third one obtains 27.59% of the optimum solutions. Compared to the selected heuristics, the three algorithms present the same computational performance level, with 29.97% average relative error, while the other four heuristics present 59.73% considering the 29 testing instances. Algorithm A1 obtains 27.56% average relative error, while the DSATUR heuristic obtains 34.30%. For computational time, algorithms A1, A2 and A3 solve the 29 instances in an average of 5.54 seconds. We avoid a computational time comparison between the generated algorithms and the existing heuristics since both are in different languages (interpreted and compiled, respectively).

### Conclusions

This paper presents the results of three new algorithms to solve the VCP. The algorithms were produced using an evolutionary computation procedure designed and implemented for that purpose that combines components of the elementary heuristics and control structures. To evolve the algorithms, 12 groups of instances selected from the DIMACS benchmark were used, and a set of 29 instances was considered to test them.

The algorithms evolved from an initial population and improved gradually as the evolutionary process occurred. The algorithm sizes stabilized at a value near the size originally given as a reference. The new algorithms are competitive in the quality of the obtained solutions to the previously existing heuristics for solving the problem; the average relative error for the testing problems is lower than the average error of the heuristics considered in the research. The new algorithms are characterized by the presence of structures similar to those in the existing heuristics for the problem.
