# Correction: Automatically Generated Algorithms for the Vertex Coloring Problem

**DOI:** 10.1371/annotation/2a72b19b-2c45-4d7e-8f3c-582eb1f57af8

**Published:** 2013-10-23

**Authors:** Carlos Contreras Bolton, Gustavo Gatica, Víctor Parada

In the introduction section, there was an error in the equation in the third sentence of the first paragraph. The sentence should begin:

"A function F: V ⟼ ℕ is defined, where ℕ is a finite set of colors ..."

This error is not present in the PDF.

There was an error in the affiliation 2 for Gustavo Gatica. Affiliation 2 should be: Escuela de Informática, Universidad Andres Bello, Santiago, Chile 

